# ROI-Based Quantitative Analysis in Contrast-Enhanced Mammography: A Feasibility Study for Reproducible Enhancement Assessment

**DOI:** 10.3390/diagnostics16162602

**Published:** 2026-08-17

**Authors:** Daniele Ugo Tari, Davide Raffaele De Lucia, Marika Santarsiere, Mariateresa Sacco, Rosalinda Santonastaso

**Affiliations:** 1 Department of Breast Imaging, District 12, Caserta Local Health Authority, 81100 Caserta, Italy; davide.delucia@aslcaserta.it (D.R.D.L.); marika.santarsiere@aslcaserta.it (M.S.); mariateresa.sacco@aslcaserta.it (M.S.); 2 Department of Economics, University of Campania “Luigi Vanvitelli”, 81043 Capua, Italy; rosalinda.santonastaso@unicampania.it

**Keywords:** contrast-enhanced mammography, enhancement assessment, breast cancer, CEM, breast density

## Abstract

**Background/Objectives:** Contrast-enhanced mammography (CEM) has demonstrated high diagnostic accuracy in breast lesion detection and characterization. However, interpretation of enhancement intensity remains largely qualitative and subject to interobserver variability. This study aimed to evaluate the feasibility and reproducibility of a standardized region-of-interest (ROI)-based quantitative approach for enhancement assessment in CEM. **Methods:** In this single-center prospective study, 22 women undergoing CEM for suspicious findings on conventional breast imaging were enrolled, yielding a total of 27 lesions. Quantitative analysis was performed on recombined mediolateral oblique images acquired in both the early and delayed post-contrast phases. Three radiologists with different levels of experience independently performed ROI measurements using two standardized ellipsoidal ROI sizes (0.1 cm^2^ and 0.2 cm^2^). ROIs were systematically positioned on the lesion, fibroglandular tissue, subcutaneous fat, and pectoralis major muscle. Relative ROI (rROI) metrics were calculated to reduce variability related to acquisition and post-processing factors. The primary normalized parameter was the lesion-to-parenchyma ratio (nROI). Histopathology served as the reference standard. **Results:** Of the 27 lesions, eight were benign (29.6%), eight were high-risk/B3 lesions (29.6%), and 11 were malignant (40.7%). Breast density categories were B in 11 lesions (40.7%), C in 13 lesions (48.1%), and D in three lesions (11.1%). Inter-reader reproducibility was good to excellent. Absolute lesion gray-scale values showed excellent agreement, with intraclass correlation coefficient (ICC) values ranging from 0.941 to 0.974, while nROI showed good reproducibility, with ICC values ranging from 0.861 to 0.894. No significant inter-reader differences were observed for normalized ratios. Comparison between ROI sizes showed small but statistically significant early phase differences for nROI, with median values of 1.009 for 0.1 cm^2^ ROIs and 1.005 for 0.2 cm^2^ ROIs (median paired difference −0.002; *p* = 0.002). Early phase nROI measured with the 0.1 cm^2^ ROI differed significantly across breast density categories, increasing from density B to D (0.996, 1.010, and 1.015, respectively; *p* = 0.026). **Conclusions:** Standardized ROI-based quantitative analysis in CEM is feasible, and normalized enhancement ratios showed good inter-reader agreement, suggesting their potential to reduce variability related to subjective visual assessment and technical acquisition factors. Consequently, the proposed method provides a reproducible methodological framework for future larger studies aimed at validating quantitative CEM metrics.

## 1. Introduction

Contrast-enhanced mammography (CEM) is an emerging imaging technique that combines 2D full-field digital mammography (2D-FFDM) with intravenous (IV) iodinated contrast administration, enabling the visualization of tumor neoangiogenesis through enhancement patterns comparable to breast magnetic resonance imaging (MRI). The technique is based on dual-energy acquisition, generating low-energy images comparable to standard mammography and high-energy images sensitive to iodine uptake. Recombined subtraction images highlight areas of abnormal vascularity and contrast enhancement, which are typically associated with malignant lesions due to increased microvascular permeability and angiogenesis [1,2,3].

CEM demonstrated high diagnostic accuracy in the detection and characterization of malignant breast lesions (95% CI 0.92 to 0.98 sensitivity; 95% CI 0.64 to 0.85 specificity), with performance similar to MRI and superior to conventional mammography [4,5]. This accuracy, close to MRI, is associated with greater accessibility and lower costs [6,7]. Furthermore, CEM provides the advantage of detecting microcalcifications, which are not reliably assessed on MRI. Therefore, it is highly recommended as a cost-effective and well-tolerated alternative for patients with contraindications to MRI [8,9].

CEM is particularly valuable in women with dense breast tissue, where sensitivity of conventional mammography is reduced due to the masking effect [10,11]. Dense breasts not only obscure lesion visibility but also represent an independent risk factor for breast cancer (BC) [12]. In this context, CEM has emerged as a promising supplemental tool by suppressing glandular background tissue through dual-energy subtraction, thus improving lesion conspicuity even in extremely dense breasts with a sensitivity of 95% and a specificity of 81% [13,14]. Despite these advantages, one of the main limitations of CEM interpretation is the frequent reliance on qualitative or subjective descriptors of enhancement intensity such as weak, moderate, or strong enhancement, which may lead to inter-observer variability [15]. Therefore, quantitative approaches using region-of-interest (ROI) analysis have been proposed to objectively assess the intensity of the enhancement [16].

A region of interest (ROI) refers to a user-defined area within an image selected for focused qualitative or quantitative analysis, typically encompassing a lesion or a representative reference tissue. ROI-based evaluation enables extraction of numerical parameters such as mean gray value, minimum and maximum intensity, standard deviation, or signal-difference-to-noise metrics, thereby transforming visual image interpretation into reproducible quantitative data [17]. In contrast-enhanced imaging modalities such as computed tomography (CT), ROIs are commonly positioned within enhancing lesions and corresponding background regions to measure enhancement and relative contrast uptake, supporting differentiation between findings. Quantitative ROI analysis may reduce subjectivity associated with purely qualitative descriptors of enhancement and improve inter-observer consistency.

Accurate placement of the region of interest (ROI), determined by the radiologist’s judgment, represents both a methodological strength and a potential limitation. On one hand, operator-guided ROI positioning allows the evaluation to be restricted to clinically relevant areas, enabling selective assessment of imaging findings and facilitating a more rapid and focused interpretation. On the other hand, this process inherently depends on operator discretion, introducing a degree of subjectivity. Both ROI placement and the quantitative parameters derived from it may be influenced by lesion characteristics, including internal homogeneity, morphology, and size. Furthermore, while densitometric measurements obtained from ROI analysis in computed tomography are relatively standardized and allow tissue differentiation largely independent of the imaging system used, similar standardization cannot be fully ensured in mammography. Variability in acquisition hardware, as well as differences in image visualization and post-processing platforms, may lead to inconsistencies in quantitative gray-value measurements. For this reason, several studies [16,17,18] have proposed the use of relative ROI-based metrics (rROI), expressed as ratios between lesion and reference-tissue gray values, in order to reduce inter-system variability and improve the robustness of quantitative assessment.

Since mammographic sensitivity decreases in dense breasts, the stratification of breast composition, according to BI-RADS 5th edition [19] (Table 1), may provide a framework to evaluate CEM diagnostic performance across different density groups.

Therefore, this feasibility study aimed to evaluate a standardized ROI-based quantitative approach for CEM enhancement assessment, comparing two predefined ROI sizes and assessing the reproducibility of absolute and normalized enhancement measurements among radiologists with different levels of experience. Given that equipment-related variability and differences in image post-processing may influence quantitative enhancement evaluation, the proposed methodology relied on predefined reference areas for ROI placement and on the calculation of rROI-derived metrics as potential standardization parameters. Additional analyses of lesion-to-reference tissue ratios across breast density categories and histopathological outcomes were performed solely for exploratory and descriptive reference purposes and were not intended to support confirmatory or diagnostic conclusions.

## 2. Materials and Methods

### 2.1. Study Design and Population

This single-center, interventional non-pharmacological study was conducted at the Department of Breast Imaging, District 12, Caserta Local Health Authority, Italy, within the organized breast cancer screening and prevention program.

We enrolled 22 women who underwent CEM, between January and December 2025, because of suspicious findings on standard imaging. A total of 27 enhancing lesions were identified, categorized according to BI-RADS 5th edition [19].

The study protocol was approved by the local Ethics Committee “Campania 3” (Resolution n. 12/2024 on 7 August 2024).

### 2.2. Imaging Protocol and ROI Analysis

**Figure 1:**
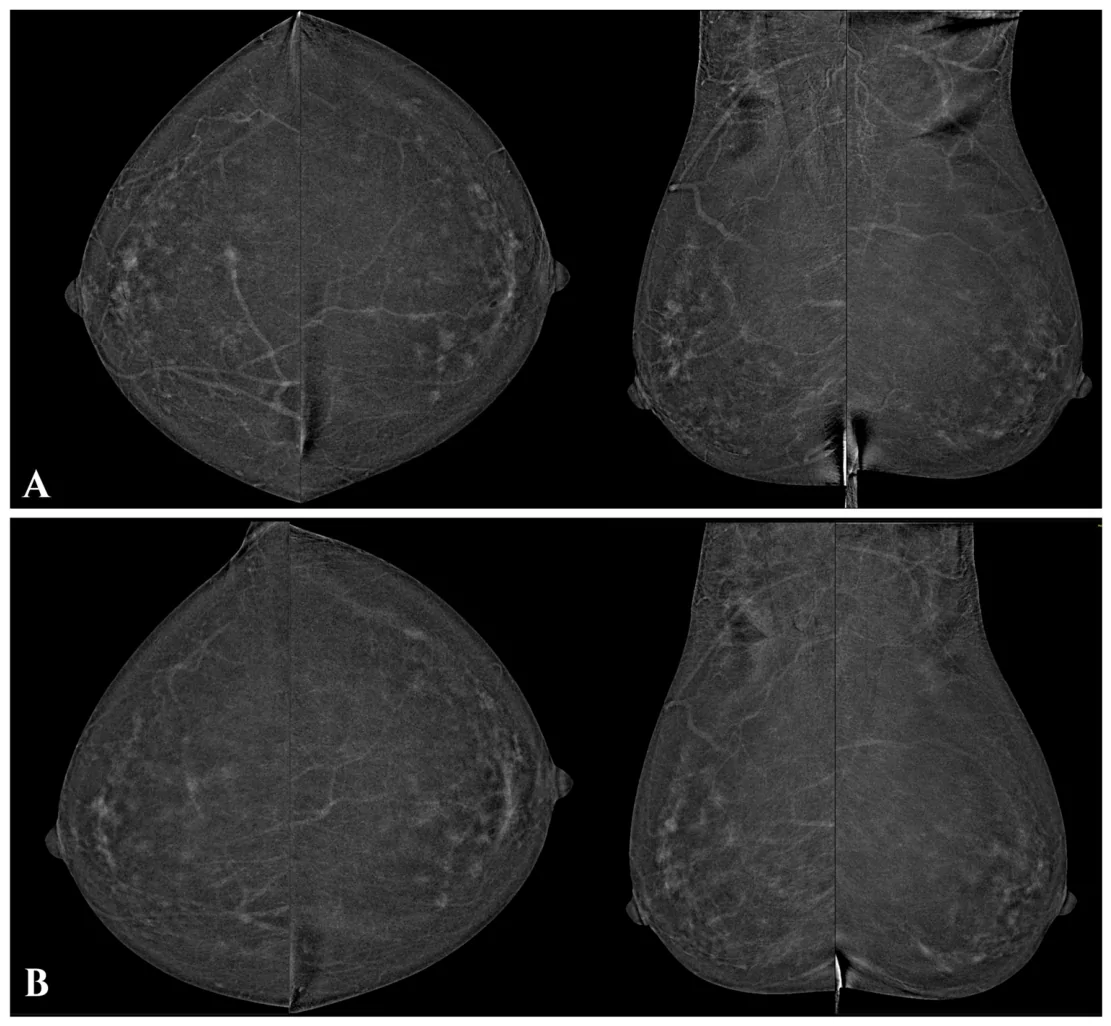
Standard acquisition protocol of contrast-enhanced mammography (CEM). (**A**) Bilateral cranio-caudal (CC) and mediolateral oblique (MLO) projections, early phase. (**B**) Bilateral cranio-caudal (CC) and mediolateral oblique (MLO) projections, delayed phase.

CEM examinations were performed using a full-field digital mammography system with dual-energy contrast-enhancement capability (Hologic Selenia Dimensions with iView software version 2.0, Hologic, Bedford, MA, USA). A non-ionic iodinated contrast agent (iohexol, 350 mg I/mL) was administered intravenously at a dose of 1.5 mL/kg with a flow rate of 3 mL/s via antecubital vein. Image acquisition started approximately 2 min after injection and was completed within 8 min, including standard craniocaudal (CC) and mediolateral oblique (MLO) projections in both the early and delayed phases (Figure 1).

Quantitative enhancement measurements were independently performed by three radiologists with different levels of experience in contrast-enhanced mammography (CEM): reader 1 (R1), with extensive experience in CEM of more than 7 years; reader 2 (R2), with intermediate experience of more than 3 years; and reader 3 (R3), with limited experience of 1 year. Measurements were performed using dedicated workstations integrated with the Picture Archiving and Communication System (PACS) and Radiology Information System (RIS).

Two ellipsoidal ROI sizes were evaluated and compared: approximately 0.1 cm^2^ and 0.2 cm^2^. The smaller ROI size was selected to allow accurate placement within the area showing the highest enhancement intensity inside the lesion. This size was considered suitable for obtaining a relatively homogeneous measurement area in both larger lesions and small enhancing foci, as well as for evaluating millimetric or non-specific findings potentially suspicious for malignancy. The larger ellipsoidal ROI was used to compare the effect of ROI size on quantitative enhancement assessment and to allow comparison with previous studies in the literature, particularly the methodology described by Rudnicki et al. [16].

For each ROI size, four standardized ROIs were systematically placed as follows (Figure 1):

- Lesion ROI (ROI-L): placed on the most enhancing solid portion of the lesion, avoiding clearly necrotic, cystic, or non-enhancing areas and ensuring that the ROI did not exceed the lesion boundaries, where possible. In large lesions, the ROI was positioned within a representative area of maximal enhancement in order to avoid underestimation of enhancement. In small findings, care was taken to avoid including adjacent parenchyma. In cases of suspicious microcalcifications without clearly visible enhancement, the ROI was still positioned in the corresponding area to detect possible subvisual contrast uptake. Parenchymal ROI (ROI-P): placed on homogeneous fibroglandular tissue, used as the reference tissue for normalization of lesion enhancement.
- Fat ROI (ROI-F): placed on homogeneous subcutaneous fat.
- Muscle ROI (ROI-M): placed on the pectoralis major muscle, considered as a relatively stable internal reference.

The main quantitative parameter used for lesion assessment was the normalized lesion enhancement ratio (nROI), calculated by normalizing the enhancement value measured within the lesion to the enhancement value measured in the surrounding fibroglandular parenchyma. This value was defined as follows:

nROI = ROI-L/ROI-P

The rationale for using this ratio was to reduce the influence of inter-patient variability in background parenchymal enhancement (BPE), breast density, contrast distribution, acquisition-related factors, and individual vascular response. In particular, in women with dense breasts or marked BPE, contrast uptake by normal fibroglandular tissue may affect both the qualitative and quantitative assessment of lesion enhancement.

Furthermore, the nROI may represent a potential standardization approach across different CEM systems and PACS systems, as absolute quantitative values may vary depending on the equipment, software, or image-processing platform used, whereas relative lesion-to-parenchyma enhancement relationships may be less affected by these differences. Additional lesion-to-fat (fROI) and lesion-to-muscle (mROI) ratios were recorded as exploratory reference parameters and calculated as follows:

fROI = ROI-L/ROI-F

mROI = ROI-L/ROI-M

**Figure 2:**
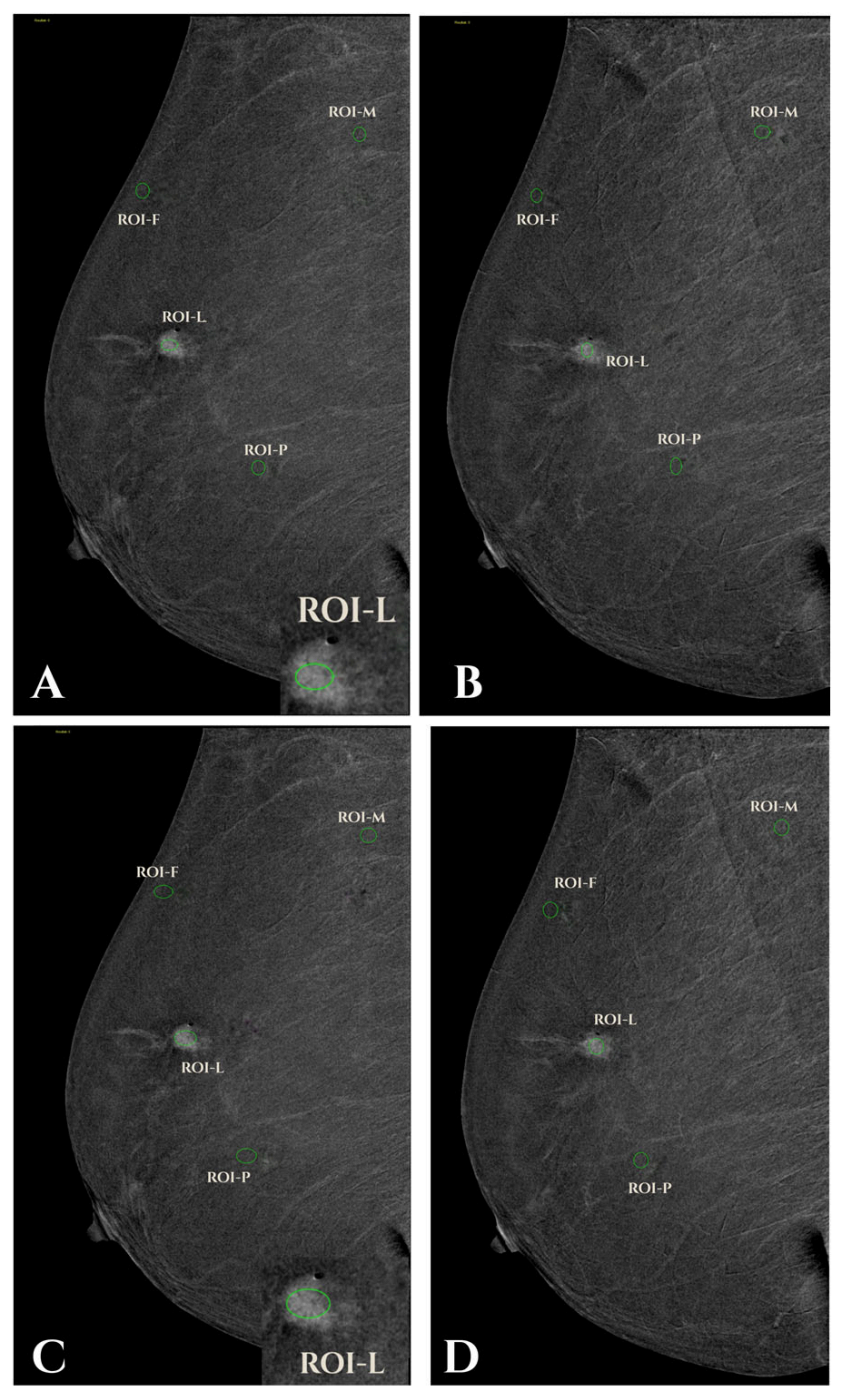
Standardized ROI placement protocol on recombined contrast-enhanced mammography. ROIs were systematically positioned on the enhancing lesion (ROI-L), fibroglandular tissue (ROI-P), subcutaneous fat (ROI-F), and pectoralis major muscle (ROI-M) to obtain normalized quantitative enhancement parameters. (**A**) right mediolateral oblique (MLO) projection, early phase, 0.1 cm^2^ ROIs with magnification of ROI-L. (**B**) right MLO projection, delayed phase, 0.1 cm^2^ ROIs. (**C**) right mediolateral oblique (MLO) projection, early phase, 0.2 cm^2^ ROIs with magnification of ROI-L. (**D**) right MLO projection, delayed phase, 0.2 cm^2^ ROIs.

The values obtained from the two ROI dimensions, approximately 0.1 cm^2^ and 0.2 cm^2^, were then compared to assess the potential impact of ROI size on normalized enhancement measurements and to evaluate the feasibility and reproducibility of this quantitative approach in CEM (Figure 2).

All measurements were performed on the MLO view, in both the early and delayed phase, as this projection provides the most consistent and reliable visualization of the pectoralis major muscle, allowing its use as a stable internal reference for ROI placement.

Breast density assessment was performed using Quantra software version 2.2.3 (Hologic, Bedford, MA, USA) with the final assessment made by the radiologist in cases of discrepancy, as supported by the literature [20,21].

Background parenchymal enhancement (BPE) was qualitatively assessed for each lesion-bearing breast by a consensus of the three radiologists according to the BI-RADS CEM categories and classified as minimal, mild, moderate, or marked.

All suspicious findings detected on CEM underwent histopathological verification by ultrasound-guided core needle biopsy or vacuum-assisted biopsy (US-CNB or US-VAB), tomosynthesis-guided vacuum-assisted biopsy (DBT-VAB), vacuum-assisted excision (VAE) or surgical excision when indicated. Histopathology served as the reference standard for lesion classification and for exploratory analysis of quantitative enhancement parameters according to pathological outcome.

### 2.3. Statistical Analysis

Statistical analysis was performed to assess the feasibility and reproducibility of the proposed ROI-based quantitative approach. Analyses were conducted separately for the two predefined ROI sizes, approximately 0.1 cm^2^ and 0.2 cm^2^, and for the early and delayed acquisition phases.

For each lesion, absolute gray-scale values were recorded for the lesion ROI, fibroglandular parenchyma ROI, fat ROI, and pectoralis muscle ROI. Three normalized ratios were calculated: nROI, fROI, and mROI. The nROI was considered the primary normalized enhancement parameter, while fROI and mROI were treated as exploratory reference ratios.

Data distribution was assessed visually and formally explored using the Shapiro–Wilk test. Since several absolute and normalized ROI-derived parameters showed non-normal or borderline distributions and given the limited sample size and feasibility design of the study, non-parametric tests were used for comparative analyses. Continuous variables were therefore summarized mainly as median and interquartile range.

Inter-reader reproducibility was assessed separately for each quantitative parameter, ROI size, and acquisition phase using a two-way mixed-effects, absolute-agreement, single-measure intraclass correlation coefficient [ICC(A,1)], with 95% confidence intervals. Lesions were treated as the measurement targets, whereas the three prespecified readers were treated as fixed raters. A single-measure ICC was selected because the intended application concerns measurements performed by an individual radiologist rather than the average of multiple readers. The Friedman test was used to assess potential systematic differences among readers. Given the absence of significant systematic inter-reader differences for the normalized parameters, lesion-level analyses were subsequently performed using the mean value of the three readers for each parameter, ROI size, and acquisition phase.

Comparisons between the two ROI sizes were performed using paired analyses. The Wilcoxon signed-rank test was used to compare normalized enhancement ratios obtained with 0.1 cm^2^ and 0.2 cm^2^ ROIs, separately for the early and delayed phases.

Agreement between nROI measurements obtained using the 0.1 cm^2^ and 0.2 cm^2^ ROIs was additionally assessed using Bland–Altman analysis, separately for the early and delayed phases. The analysis was performed using lesion-level mean values across the three readers. Bias was calculated as the mean difference between measurements obtained with the 0.2 cm^2^ and 0.1 cm^2^ ROIs, and 95% limits of agreement were calculated as the mean difference ± 1.96 standard deviations of the paired differences. The presence of proportional bias was explored by linear regression of the paired differences against their mean.

Exploratory analyses according to histopathological outcome and breast density category were performed using non-parametric tests. Differences among benign, high-risk/B3, and malignant lesions were assessed using the Kruskal–Wallis test, while malignant versus non-malignant comparisons were performed using the Mann–Whitney U test. Given the feasibility design and limited sample size, these analyses were considered exploratory and no diagnostic threshold was derived. A two-sided *p*-value < 0.05 was considered statistically significant. Statistical analyses were performed by using STATA 13 (StataCorp LLC, College Station, TX, USA).

## 3. Results

### 3.1. Study Population

**Figure 3:**
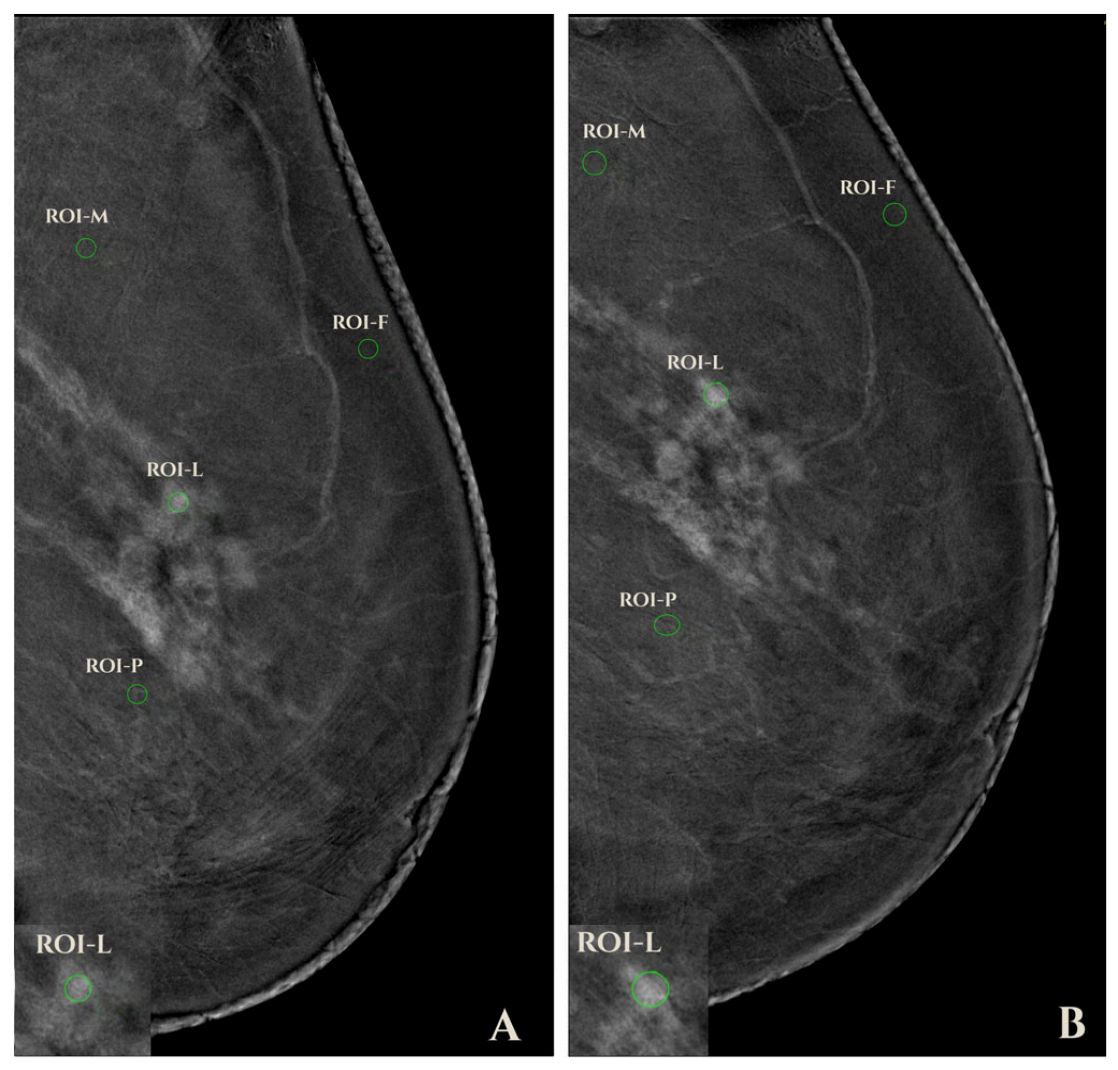
Contrast-enhanced mammography demonstrating, in the upper quadrant of the left breast, a regional non-mass-like enhancement (NMLE) with early and heterogeneous contrast uptake. Ellipsoidal regions of interest (ROIs; area = 0.1 cm^2^) were positioned within the area of maximal enhancement of the lesion (ROI-L), in the background fibroglandular tissue (ROI-P), subcutaneous fat (ROI-F), and pectoralis major muscle (ROI-M). (**A**) Early phase left mediolateral oblique (MLO) image with magnification of ROI-L. (**B**) Delayed phase left mediolateral oblique (MLO) image with magnification of ROI-L. Histopathological result of US-CNB: invasive ductal carcinoma, no special type.

**Figure 4:**
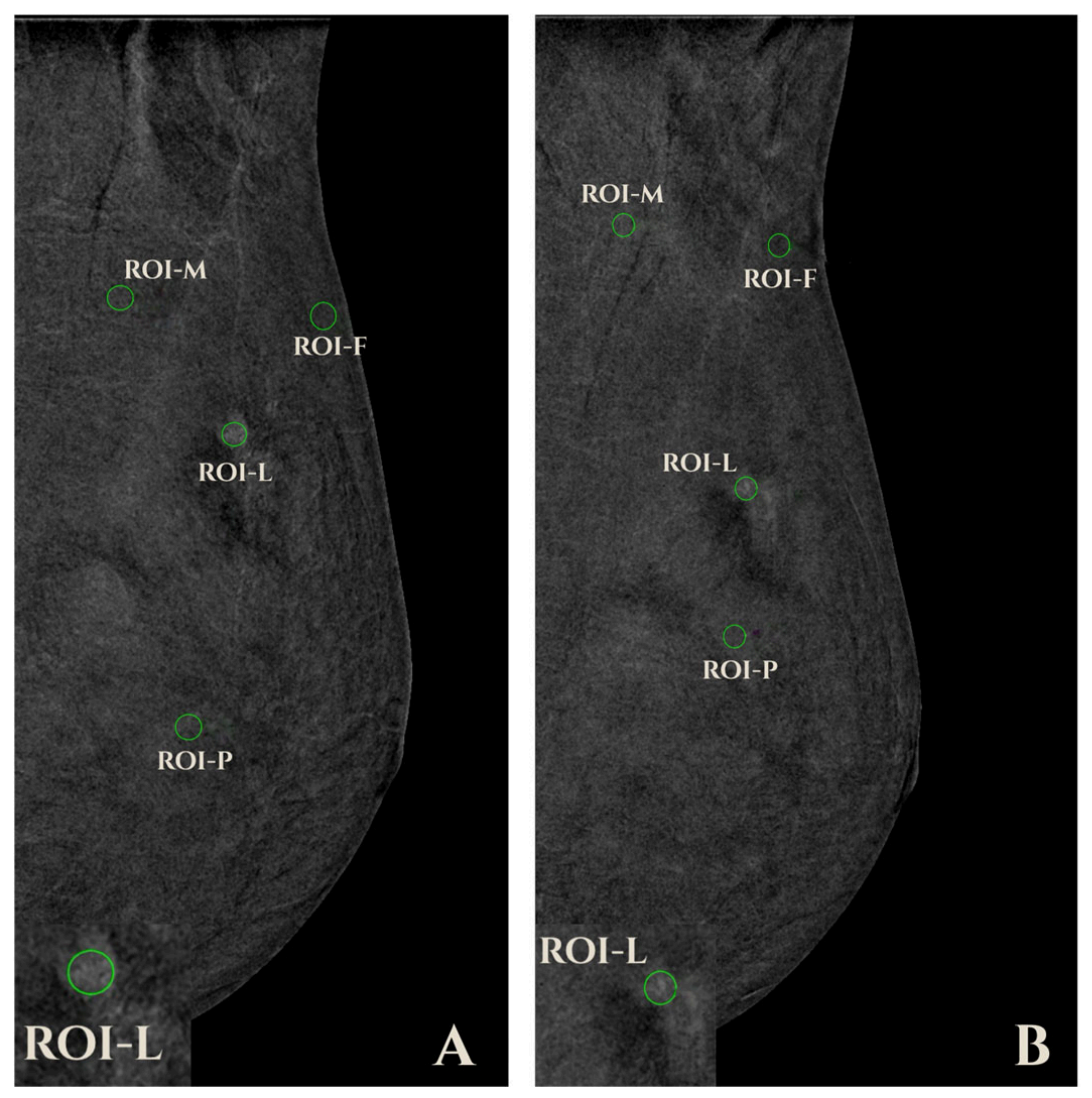
Contrast-enhanced mammography demonstrating early and heterogeneous enhancement corresponding to a cluster of microcalcifications in the upper outer quadrant of the left breast. Ellipsoidal regions of interest (ROIs; area = 0.1 cm^2^) were placed on the enhancing lesion (ROI-L), background fibroglandular tissue (ROI-P), subcutaneous fat (ROI-F), and pectoralis major muscle (ROI-M). (**A**) Left mediolateral oblique (MLO) view, early phase with magnification of ROI-L. (**B**) Left MLO view, delayed phase with magnification of ROI-L. Histopathological result of US-VAB: invasive ductal carcinoma, no special type.

A total of 22 women were included, with 27 enhancing lesions analyzed on a lesion-level basis. The median patient age was 58 years (interquartile range [IQR], 51–61 years; range, 45–77 years). No lesion was observed in ACR breast density category A; 11 lesions (40.7%) were observed in category B, 13 (48.1%) in category C, and 3 (11.1%) in category D. Background parenchymal enhancement was predominantly low in the study cohort. At lesion level, BPE was minimal in 16 cases (59.3%), mild in 4 (14.8%), moderate in 3 (11.1%), and marked in 4 (14.8%). Overall, minimal or mild BPE was observed in 74.1% of lesions. Given the limited sample size and the small number of cases within individual BPE categories, no subgroup analysis was performed, and these data should be considered descriptive only. Histopathology showed 8 benign lesions (29.6%), 8 high-risk/B3 lesions (29.6%), and 11 malignant lesions (40.7%) (Figure 3 and Figure 4). The main characteristics of the study population and lesions are summarized in Table 2.

### 3.2. Distribution of ROI-Derived Parameters and Inter-Reader Reproducibility

The distribution of absolute and normalized ROI-derived parameters was visually assessed and formally explored using the Shapiro–Wilk test. Several parameters showed non-normal or borderline distributions, particularly among delayed-phase measurements and normalized ratios (see Supplementary Materials, Table S1). Therefore, quantitative variables were mainly summarized using median and IQR, and non-parametric tests were used for comparative analyses.

Inter-reader reproducibility was evaluated among the three radiologists for absolute lesion gray-scale values and normalized enhancement ratios, separately for the two ROI sizes and acquisition phases. Overall, the proposed ROI-based quantitative approach showed good to excellent inter-reader reproducibility (Table 3). Absolute lesion gray-scale values showed excellent agreement, with ICC values ranging from 0.941 to 0.974 across ROI sizes and acquisition phases. The primary normalized parameter, nROI, showed good reproducibility, with ICC values ranging from 0.861 to 0.894. The additional exploratory normalized ratios also demonstrated good to excellent reproducibility, with ICC values ranging from 0.867 to 0.953 for fROI and from 0.889 to 0.915 for mROI.

The Friedman test did not show significant systematic differences among readers for the normalized parameters. A significant inter-reader difference was observed only for the absolute lesion gray-scale value measured with the 0.2 cm^2^ ROI in the delayed phase; however, this difference was not observed for nROI, fROI, or mROI. Therefore, subsequent lesion-level analyses were performed using the mean value of the three readers for each parameter, ROI size, and acquisition phase.

### 3.3. Comparison Between ROI Sizes

The effect of ROI size was evaluated by comparing measurements obtained with approximately 0.1 cm^2^ and 0.2 cm^2^ ROIs on the same lesions (Table 4).

In the early phase, statistically significant differences were observed between the two ROI sizes for absolute lesion gray-scale values and normalized ratios. For nROI, the median value was 1.009 for the 0.1 cm^2^ ROI and 1.005 for the 0.2 cm^2^ ROI, with a median paired difference of −0.002 (*p* = 0.002). Similar statistically significant but numerically small differences were observed for fROI and mROI in the early phase.

In the delayed phase, no statistically significant differences were observed between the two ROI sizes for absolute lesion gray-scale values or normalized ratios. For nROI, the median value was 1.004 for the 0.1 cm^2^ ROI and 1.002 for the 0.2 cm^2^ ROI (*p* = 0.141).

**Figure 5:**
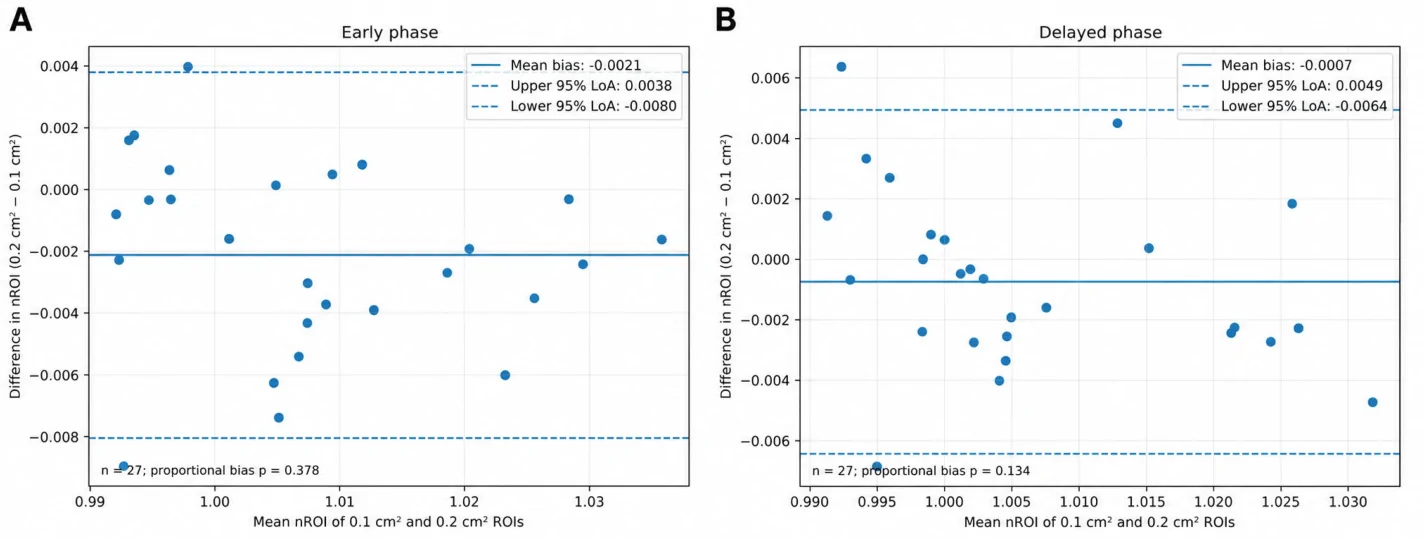
Bland–Altman analysis of agreement between nROI measurements obtained using 0.1 cm^2^ and 0.2 cm^2^ ROIs. Differences were calculated as 0.2 cm^2^ minus 0.1 cm^2^ measurements using lesion-level mean values across the three readers. Each blue dot represents an individual observation. The solid horizontal line represents the mean bias, while the dashed lines indicate the 95% limits of agreement (LoA). (**A**) Early phase. (**B**) Delayed phase. No significant proportional bias was observed in either phase.

Bland–Altman analysis showed a small negative bias between ROI sizes in the early phase. The mean difference between the 0.2 cm^2^ and 0.1 cm^2^ ROIs was −0.0021, with 95% limits of agreement ranging from −0.0080 to 0.0038. In the delayed phase, the mean bias was −0.0007, with 95% limits of agreement ranging from −0.0064 to 0.0049. No significant proportional bias was observed in either the early (*p* = 0.378) or delayed phase (*p* = 0.134) (Figure 5).

These findings suggest that ROI size may slightly influence quantitative enhancement measurements, particularly in the early phase, although the magnitude of the observed differences was small.

### 3.4. Histopathological Outcome and Breast Density

Exploratory analyses were performed to evaluate whether nROI differed according to histopathological outcome (Table 5) and ACR breast density category (Table 6). No statistically significant differences in nROI were observed among benign, high-risk/B3, and malignant lesions for either ROI size or acquisition phase (Table 5). Additional comparisons between malignant and non-malignant lesions were also not significant (Mann–Whitney U test *p* range: 0.312–0.639). Therefore, no diagnostic threshold was derived from the present feasibility cohort.

Exploratory analysis according to breast density (Table 6) showed a significant difference in early phase nROI measured with the 0.1 cm^2^ ROI across ACR density categories, with median values of 0.996 in category B, 1.010 in category C, and 1.015 in category D (*p* = 0.026; Table 6). A similar trend was observed using the 0.2 cm^2^ ROI, although it did not reach statistical significance (*p* = 0.066). No significant density-related differences were observed in the delayed phase. Given the limited sample size, particularly in density category D, these findings should be considered exploratory.

### 3.5. Summary of Main Findings

The proposed standardized ROI-based quantitative approach was feasible and reproducible for CEM enhancement assessment. Inter-reader agreement was good to excellent across ROI sizes and acquisition phases, including for the primary normalized parameter, nROI. The absence of significant systematic inter-reader differences for normalized ratios supports the reliability of the predefined ROI placement protocol, even among radiologists with different levels of CEM experience.

The comparison between ROI sizes showed small but statistically significant differences in the early phase, whereas delayed-phase measurements were comparable between the 0.1 cm^2^ and 0.2 cm^2^ ROIs. These findings suggest that ROI size may have an influence on early enhancement quantification, while delayed normalized measurements appear more stable.

Exploratory analyses according to histopathological outcomes did not demonstrate significantly higher normalized enhancement values in malignant lesions compared with benign or high-risk/B3 findings. Conversely, exploratory density-based analyses showed a possible association between early phase nROI and breast density. These findings require validation in larger samples.

Overall, our results indicate that the proposed quantitative CEM protocol is methodologically feasible and reproducible.

## 4. Discussion

In routine clinical practice, CEM interpretation is based on qualitative evaluation of lesion morphology, enhancement characteristics and BPE. The introduction of objective and reproducible imaging biomarkers could be useful to reduce the subjectivity of visual assessment and to support a more standardized interpretation of images. A relevant example is represented by breast density assessment. The introduction of its quantitative evaluation, subsequently integrated with AI-driven qualitative assessment tools, has substantially supported radiologists in patient evaluation and risk stratification [20,21]. In particular, these approaches have improved the identification of women with dense breasts, a subgroup characterized by reduced mammographic sensitivity due to lesion masking and by an increased risk of developing breast cancer [20,22,23].

The present study should be interpreted as a preliminary methodological step toward future larger studies aimed at validating quantitative enhancement metrics and defining clinically relevant diagnostic cut-offs.

In this context, the central parameter of the proposed method is the normalized lesion-to-parenchyma enhancement ratio, nROI, calculated as the ratio between lesion enhancement and fibroglandular parenchymal enhancement. This parameter was selected because it directly normalizes lesion enhancement to the surrounding reference tissue and may therefore reduce the influence of inter-patient variability, BPE, breast density, acquisition parameters, and post-processing factors.

By contrast, lesion-to-fat (fROI) and lesion-to-muscle (mROI) ratios were included as exploratory reference metrics. Their role was mainly to provide internal methodological validation and to test the consistency of the measurement strategy across different anatomical reference tissues. They should not be considered equivalent to nROI as primary quantitative parameters. From a practical perspective, nROI remains the most relevant and necessary metric in the present protocol, because it is directly related to the lesion-to-parenchyma relationship on recombined CEM images.

The main finding of this study is that a standardized ROI-based quantitative approach can be applied consistently by radiologists with different levels of CEM experience. Inter-reader agreement was good to excellent for absolute lesion gray-scale values and remained good for normalized enhancement ratios, particularly nROI. Moreover, no significant inter-reader differences were observed for normalized metrics. These findings suggest that predefined ROI placement criteria may reduce operator-dependent variability and provide a reproducible basis for quantitative CEM assessment.

Previous quantitative CEM studies [16,17,18,24] reported promising diagnostic findings but used heterogeneous and incompletely standardized ROI methodologies. Some authors have suggested that quantitative CEM-derived parameters may contribute to the differentiation between benign and malignant breast lesions. Deng et al. [25] reported stronger enhancement in malignant lesions than in benign lesions and evaluated relative enhancement between early and delayed phases; Rudnicki et al. [16] found a significant correlation between quantitative enhancement assessment and histopathology; Lv et al. [17] reported that quantitative enhancement intensity on CESM may provide diagnostic information and showed only moderate agreement between CESM enhancement patterns and DCE-MRI; and Xu et al. [18] further explored multiphase quantitative CEM and found that early phase quantitative parameters showed relevant diagnostic performance, whereas the added delayed phase provided limited improvement. These studies support the biological and diagnostic potential of quantitative enhancement assessment, but they were mainly focused on diagnostic performance rather than on reproducibility of ROI placement and methodological standardization, and this aspect may have influenced the results. Our study addresses this potential issue.

Before quantitative CEM parameters can be used as diagnostic biomarkers, the method used to obtain them must be reproducible. ROI positioning is a critical issue in quantitative enhancement assessment. Small ROIs may better sample the most enhancing portion of a lesion and may be particularly useful in small lesions or focal enhancements. However, they may also be more sensitive to minor differences in placement. Larger or whole-lesion ROIs may better reflect lesion heterogeneity, but they can include necrotic, cystic, poorly vascularized, or non-enhancing components, potentially resulting in underestimation of lesion enhancement and subsequent misinterpretation of quantitative findings. Therefore, standardized sampling criteria are essential before quantitative CEM metrics can be reliably compared across readers, centers, and imaging platforms.

Future studies should evaluate whether specific recommendations are needed for heterogeneous masses, rim-enhancing lesions, non-mass-like enhancement, very small findings, and lesions containing necrotic or non-enhancing components.

The comparison between ROI sizes showed broadly comparable results between 0.1 cm^2^ and 0.2 cm^2^ ROIs. Nevertheless, small but statistically significant differences were observed in the early phase, whereas delayed-phase measurements were more stable across ROI dimensions. Bland–Altman analysis confirmed a small systematic tendency toward lower nROI values with the 0.2 cm^2^ ROI in the early phase, whereas the delayed-phase bias was close to zero. The limits of agreement were relatively narrow around zero; however, their clinical acceptability cannot be established because clinically meaningful tolerance limits for nROI have not yet been defined.

These findings could be methodologically relevant. The early phase is particularly important in contrast-enhanced breast imaging because malignant lesions are typically characterized by neoangiogenesis, increased microvascular permeability, and rapid contrast uptake. These mechanisms make early iodine enhancement a key component of lesion conspicuity and diagnostic interpretation in CEM [18,26].

This aspect also highlights an important difference between CEM and breast MRI. Dynamic contrast-enhanced MRI (DCE-MRI) allows repeated acquisitions over time and the generation of time–intensity curves, usually classified as progressive, plateau, or washout patterns. These kinetic curves provide information on both early uptake and delayed enhancement behavior. In CEM, however, image acquisition is based on a limited number of projections obtained at predefined time points after iodinated contrast administration. Therefore, CEM does not provide continuous time–intensity curves in the same way as DCE-MRI. Multiphase or delayed CEM protocols may approximate kinetic assessment, but they are influenced by acquisition order, breast compression, projection differences, radiation dose, and protocol variability [27]. In this context, a standardized mathematical ROI-based calculation may be particularly useful, because it provides an objective way to quantify enhancement within the technical constraints of mammography.

**Figure 6:**
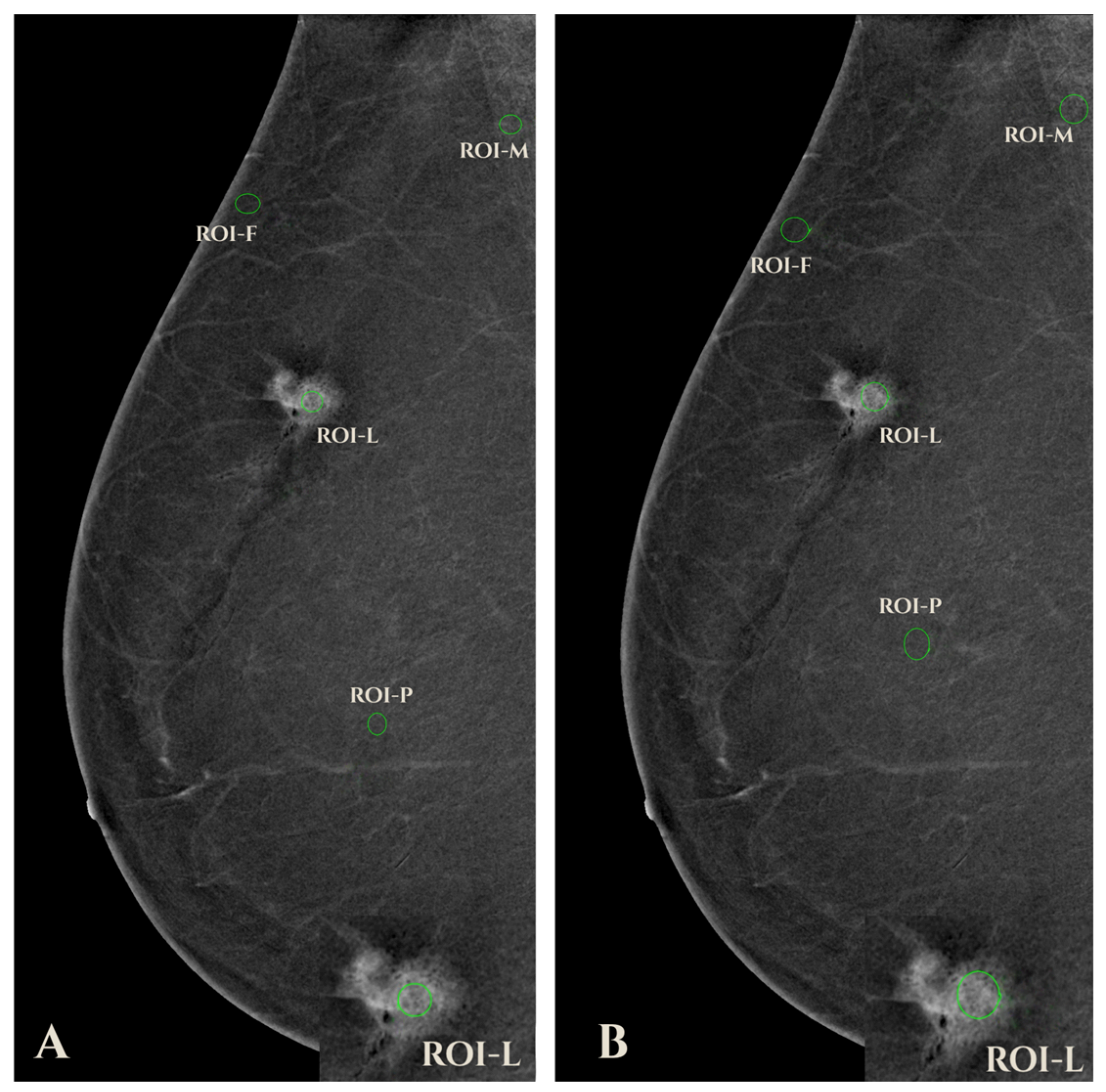
Contrast-enhanced mammography showing a mass-like enhancing lesion (MLE) involving the upper quadrants of the right breast, with intense, early, and heterogeneous enhancement. Enhancement heterogeneity reflects the presence of associated microcalcifications and intratumoral necrotic areas. (**A**) Early phase right MLO image with magnification of ROI-L. Ellipsoidal ROIs (0.1 cm^2^ each) were positioned within the region of greatest enhancement, avoiding necrotic and heterogeneous areas. (**B**) Early phase right MLO image with magnification of ROI-L. Ellipsoidal ROIs (0.2 cm^2^ each) were positioned within the region of greatest enhancement, including necrotic and heterogeneous areas. Histopathological result of US-VAB: invasive ductal carcinoma, no special type.

The observed early phase sensitivity to ROI size may therefore have practical implications. Since early enhancement is closely related to the vascular behavior of lesions [28], even small differences in ROI placement may affect quantitative values (Figure 6). This supports the need for a carefully standardized ROI protocol, especially when quantitative CEM metrics are intended for future diagnostic threshold definition, density-adjusted analyses, radiomics, or artificial intelligence applications.

In this study, nROI did not significantly differ among benign, high-risk, and malignant lesions. Nevertheless, the current cohort was not powered to establish diagnostic thresholds or to determine whether nROI can reliably differentiate benign from malignant lesions, due to the limited sample size, and the inclusion of clinically suspicious findings with potential overlapping enhancement patterns.

The observed association between early phase nROI and breast density is very interesting but deserves further investigations. Early phase nROI measured with the 0.1 cm^2^ ROI showed a slight increase across increasing breast density categories. This finding may suggest that breast density influences normalized lesion-to-parenchyma enhancement assessment. However, the number of lesions in density category D was very limited, and no definitive conclusions can be drawn. Future studies should specifically evaluate whether breast density modifies quantitative enhancement behavior and whether density-adjusted nROI cut-offs may improve diagnostic performance.

Another relevant aspect is the potential impact of BPE on quantitative CEM assessment. BPE reflects contrast uptake by normal fibroglandular tissue and may vary according to hormonal status, breast density, vascularity, timing of acquisition, and individual contrast kinetics [29,30]. In qualitative CEM interpretation, prominent BPE may reduce lesion conspicuity or complicate the visual assessment of enhancement intensity, particularly in dense breasts [31]. From a quantitative perspective, BPE may also influence absolute lesion gray-scale values and may contribute to inter-patient variability. For this reason, normalization of lesion enhancement to fibroglandular parenchyma, as performed through nROI, is methodologically relevant. By expressing lesion enhancement relative to the background parenchymal signal, nROI may partially account for individual differences in BPE and provide a more reproducible lesion-to-background measurement than absolute enhancement values alone. However, this approach also implies that marked or heterogeneous BPE could affect the denominator of the ratio and therefore influence nROI values. This represents an important issue for future studies, which should evaluate whether BPE modifies quantitative enhancement behavior and whether BPE-adjusted or density-adjusted nROI thresholds may improve lesion characterization in CEM.

Overall, these findings support the concept that quantitative CEM analysis may represent an intermediate step between qualitative visual interpretation and advanced computational imaging. A reproducible ROI-based method is a necessary prerequisite before quantitative enhancement parameters can be tested in larger cohorts, compared across institutions, or integrated into radiomics and artificial intelligence workflows.

This study has several limitations. The sample size and the number of lesions in some breast density categories were limited, and this limits the ability to draw conclusions regarding diagnostic cut-offs, benign–malignant differentiation, lesion subtype characterization, or density-specific enhancement behavior. Although ROI placement was standardized, anatomical variability, lesion heterogeneity, and differences in enhancement kinetics may still affect quantitative measurements. Measurements were restricted to the MLO projection to ensure consistent availability of the pectoralis muscle as an internal reference. This methodological choice does not reproduce comprehensive clinical CEM interpretation, which relies on both CC and MLO projections. Future studies should evaluate whether lesion-based quantitative measurements can be reproducibly performed on the projection in which the lesion is best visualized. Finally, this was a single-center study performed on a single CEM platform; therefore, the proposed method requires validation across different vendors, workstations, image-processing systems, and PACS environments.

Future studies should apply this standardized ROI-based protocol to larger and more balanced cohorts, including adequate representation of different lesions across breast density categories. In particular, nROI should be prospectively evaluated to determine whether its value, calculation method, or density and/or BPE-adjusted interpretation can improve lesion characterization at CEM. Larger multicenter studies are also needed to assess inter-vendor reproducibility and explore the integration of ROI-based assessment into radiomics and artificial intelligence models [32].

## 5. Conclusions

This feasibility study supports the reproducibility and technical applicability of a standardized ROI-based quantitative method for CEM enhancement assessment. The proposed approach appears suitable as a methodological foundation for future larger studies aimed at validating quantitative enhancement metrics in personalized breast imaging pathways.

## Figures and Tables

**Table 1:** Breast density classification according to BI-RADS 5th edition [19].

Breast Density Category	Definition	Description	Implication
A	Almost entirely fatty	The breast is almost entirely composed of fatty tissue, with minimal fibro-glandular tissue.	High mammographic sensitivity.
B	Scattered areas of fibroglandular density	Scattered areas of fibroglandular tissue are present, but most of the breast is fatty.	Mammographic sensitivity is generally preserved.
C	Heterogeneously dense	The breast contains heterogeneous areas of dense fibroglandular tissue, which may obscure small masses.	Reduced mammographic sensitivity due to a masking effect.
D	Extremely dense	The breast is almost entirely composed of dense fibroglandular tissue.	Markedly reduced mammographic sensitivity; higher masking effect.

**Table 2:** Study population and lesion characteristics.

Characteristic	Value
Patients, *n*	22
Enhancing lesions analyzed, *n*	27
Age, years, median (IQR)	58 (51–61)
Age, years, range	45–77
ACR breast density category A, *n* (%)	0 (0.0%)
ACR breast density category B, *n* (%)	11 (40.7%)
ACR breast density category C, *n* (%)	13 (48.1%)
ACR breast density category D, *n* (%)	3 (11.1%)
BI-RADS 2, *n* (%)	1 (3.7%)
BI-RADS 3, *n* (%)	11 (40.7%)
BI-RADS 4, *n* (%)	2 (7.4%)
BI-RADS 4A, *n* (%)	3 (11.1%)
BI-RADS 4B, *n* (%)	2 (7.4%)
BI-RADS 4C, *n* (%)	3 (11.1%)
BI-RADS 5, *n* (%)	5 (18.5%)
Benign lesions, *n* (%)	8 (29.6%)
High-risk/B3 lesions, *n* (%)	8 (29.6%)
Malignant lesions, *n* (%)	11 (40.7%)

Data are reported on a lesion-level basis unless otherwise specified. IQR: interquartile range; ACR: American College of Radiology; BI-RADS: Breast Imaging Reporting and Data System [19].

**Table 3:** Inter-reader reproducibility of absolute and normalized ROI-derived parameters.

ROI Size (cm^2^)	Phase	Parameter	ICC (95% CI)	Friedman *p*
0.1	Early	ROI-L gray value	0.973 (0.937–0.989)	0.594
0.1	Early	nROI	0.879 (0.778–0.927)	0.459
0.1	Early	fROI	0.948 (0.895–0.972)	0.054
0.1	Early	mROI	0.889 (0.780–0.947)	0.627
0.1	Delayed	ROI-L gray value	0.941 (0.846–0.986)	0.932
0.1	Delayed	nROI	0.861 (0.729–0.925)	0.936
0.1	Delayed	fROI	0.867 (0.738–0.937)	0.134
0.1	Delayed	mROI	0.889 (0.768–0.952)	0.462
0.2	Early	ROI-L gray value	0.974 (0.943–0.988)	0.656
0.2	Early	nROI	0.874 (0.750–0.939)	0.264
0.2	Early	fROI	0.936 (0.824–0.983)	0.06
0.2	Early	mROI	0.915 (0.811–0.962)	0.156
0.2	Delayed	ROI-L gray value	0.968 (0.925–0.986)	0.007
0.2	Delayed	nROI	0.894 (0.807–0.934)	1.0
0.2	Delayed	fROI	0.953 (0.895–0.974)	0.087
0.2	Delayed	mROI	0.914 (0.776–0.967)	0.478

ICC: intraclass correlation coefficient; CI: confidence interval; ROI-L: lesion ROI; nROI: ROI-L/ROI-P; fROI: ROI-L/ROI-F; mROI: ROI-L/ROI-M. Friedman *p*-values assess systematic differences among readers. ICC values refer to a two-way mixed-effects, absolute-agreement, single-measure model [ICC(A,1)].

**Table 4:** Paired comparison between 0.1 cm^2^ and 0.2 cm^2^ ROIs.

Phase	Parameter	0.1 cm^2^ Median (IQR)	0.2 cm^2^ Median (IQR)	Median Paired Difference (0.2 − 0.1)	Wilcoxon *p*
Early	ROI-L gray value	2101 (2082–2124)	2095 (2080–2121)	−3.000	<0.001
Early	nROI	1.009 (0.996–1.017)	1.005 (0.996–1.015)	−0.002	0.002
Early	fROI	1.017 (1.007–1.026)	1.014 (1.006–1.025)	−0.001	<0.001
Early	mROI	1.005 (0.996–1.014)	1.003 (0.994–1.012)	−0.001	<0.001
Delayed	ROI-L gray value	2097 (2081–2112)	2094 (2080–2111)	−2.000	0.086
Delayed	nROI	1.004 (0.998–1.013)	1.002 (0.998–1.015)	−0.001	0.141
Delayed	fROI	1.014 (1.007–1.021)	1.012 (1.008–1.020)	−0.000	0.470
Delayed	mROI	1.005 (0.995–1.009)	1.003 (0.996–1.006)	−0.000	0.427

Values are lesion-level means across the three readers and are reported as median (IQR). Paired differences were calculated as 0.2 cm^2^ minus 0.1 cm^2^. ROI-L: lesion ROI; nROI: ROI-L/ROI-P; fROI: ROI-L/ROI-F; mROI: ROI-L/ROI-M.

**Table 5:** Exploratory nROI analysis according to histopathological outcome.

ROI Size (cm^2^)	Phase	Benign	High-Risk/B3	Malignant	Kruskal–Wallis *p*
0.1	Early	1.013 (1.005–1.023)	1.001 (0.996–1.009)	1.009 (0.998–1.024)	0.4
0.1	Delayed	1.004 (1.000–1.014)	1.000 (0.997–1.006)	1.006 (0.998–1.025)	0.318
0.2	Early	1.009 (1.002–1.020)	1.001 (0.997–1.005)	1.005 (0.997–1.023)	0.375
0.2	Delayed	1.002 (1.000–1.016)	1.000 (0.997–1.003)	1.003 (0.998–1.024)	0.375

nROI values are lesion-level means across the three readers and are reported as median (IQR). nROI: ROI-L/ROI-P.

**Table 6:** Exploratory nROI analysis according to ACR breast density category.

ROI Size (cm^2^)	Phase	Density B	Density C	Density D	Kruskal–Wallis *p*
0.1	Early	0.996 (0.993–1.008)	1.010 (1.005–1.021)	1.015 (1.012–1.026)	0.026
0.1	Delayed	1.000 (0.994–1.005)	1.006 (1.000–1.023)	1.008 (1.005–1.017)	0.204
0.2	Early	1.000 (0.994–1.004)	1.005 (1.001–1.019)	1.011 (1.010–1.023)	0.066
0.2	Delayed	0.999 (0.996–1.003)	1.003 (1.000–1.020)	1.007 (1.004–1.017)	0.206

nROI values are lesion-level means across the three readers and are reported as median (IQR). ACR: American College of Radiology; nROI: ROI-L/ROI-P.

## Data Availability

The dataset can be found on saniarp.it, in the Caserta LHA reporting database, and in the register of our daily activities.

## Supplementary Materials

The following supporting information can be downloaded at https://www.mdpi.com/article/10.3390/diagnostics16162602/s1, Table S1: Distribution assessment of absolute and normalized ROI-derived quantitative parameters.

## Abbreviations

The following abbreviations are used in this manuscript:

2D-FFDM	two-dimensional full-field digital mammography
ACR	American College of Radiology
AI	artificial intelligence
BC	breast cancer
BI-RADS	Breast Imaging Reporting and Data System
BPE	background parenchymal enhancement
CC	craniocaudal
CEM	contrast-enhanced mammography
CESM	contrast-enhanced spectral mammography
CI	confidence interval
CT	computed tomography
DBT-VAB	digital breast tomosynthesis-guided vacuum-assisted biopsy
DCE-MRI	dynamic contrast-enhanced magnetic resonance imaging
fROI	lesion-to-fat ratio
ICC	intraclass correlation coefficient
IQR	interquartile range
IV	intravenous
MLO	mediolateral oblique
mROI	lesion-to-muscle ratio
MRI	magnetic resonance imaging
nROI	normalized lesion-to-parenchyma ratio
PACS	Picture Archiving and Communication System
R1	reader 1
R2	reader 2
R3	reader 3
RIS	Radiology Information System
ROI	region of interest
ROI-F	fat ROI
ROI-L	lesion ROI
ROI-M	muscle ROI
ROI-P	parenchymal ROI
rROI	relative ROI
US-CNB	ultrasound-guided core needle biopsy
US-VAB	ultrasound-guided vacuum-assisted biopsy
VAE	vacuum-assisted excision
