# Supplementary material for: ROI-Based Quantitative Analysis in Contrast-Enhanced Mammography: A Feasibility Study for Reproducible Enhancement Assessment
Source: Diagnostics (Basel). 2026 Aug 17;16(16):2602. doi: 10.3390/diagnostics16162602 (PMC13512133; doi:10.3390/diagnostics16162602)
Supplement: Supplementary file 1 [file diagnostics-16-02602-s001.zip › diagnostics-4417247-supplementary.pdf]

Supplementary Materials

Table S1. Distribution assessment of absolute and normalized ROI-derived quantitative parameters.

Data distribution was preliminarily assessed at lesion level using the mean value of the three readers for each parameter, ROI size, and acquisition phase. The Shapiro-Wilk test was used to formally explore normality. A *p*-value < 0.05 was considered indicative of deviation from normal distribution; *p*-values between 0.05 and 0.10 were classified as borderline. This supplementary assessment supports the use of non-parametric tests for the comparative analyses reported in the main manuscript.

ROI size (cm^2)	Phase	Parameter	*n*	Mean ± SD	Median (IQR)	Range	Shapiro–Wilk *p*	Distribution
0.1	Early	ROI-L	27	2103.9 ± 27.4	2101.3 (2082.3–2123.8)	2064.3–2162.7	0.252	Compatible with normality
0.1	Early	nROI	27	1.0089 ± 0.0132	1.0089 (0.9963–1.0173)	0.9923–1.0366	0.050	Non-normal
0.1	Early	fROI	27	1.0184 ± 0.0127	1.0172 (1.0065–1.0263)	1.0019–1.0483	0.167	Compatible with normality
0.1	Early	mROI	27	1.0064 ± 0.0125	1.0053 (0.9959–1.0142)	0.9895–1.0381	0.096	Borderline
0.1	Delayed	ROI-L	27	2100.3 ± 25.5	2096.7 (2081.3–2111.8)	2065.7–2161.0	0.046	Non-normal
0.1	Delayed	nROI	27	1.0067 ± 0.0123	1.0035 (0.9985–1.0128)	0.9892–1.0341	0.048	Non-normal
0.1	Delayed	fROI	27	1.0162 ± 0.0110	1.0144 (1.0075–1.0213)	1.0000–1.0420	0.150	Compatible with normality
0.1	Delayed	mROI	27	1.0046 ± 0.0122	1.0046 (0.9951–1.0093)	0.9879–1.0331	0.100	Borderline
0.2	Early	ROI-L	27	2099.6 ± 26.1	2095.3 (2079.5–2121.2)	2064.0–2151.3	0.090	Borderline
0.2	Early	nROI	27	1.0067 ± 0.0127	1.0049 (0.9965–1.0147)	0.9882–1.0350	0.166	Compatible with normality
0.2	Early	fROI	27	1.0163 ± 0.0123	1.0142 (1.0060–1.0248)	1.0010–1.0440	0.033	Non-normal
0.2	Early	mROI	27	1.0041 ± 0.0119	1.0032 (0.9945–1.0121)	0.9866–1.0323	0.407	Compatible with normality
0.2	Delayed	ROI-L	27	2099.1 ± 23.9	2094.3 (2079.8–2111.2)	2069.3–2157.7	0.019	Non-normal
0.2	Delayed	nROI	27	1.0059 ± 0.0114	1.0021 (0.9978–1.0152)	0.9916–1.0294	0.005	Non-normal
0.2	Delayed	fROI	27	1.0159 ± 0.0110	1.0122 (1.0078–1.0200)	1.0011–1.0430	0.016	Non-normal
0.2	Delayed	mROI	27	1.0042 ± 0.0110	1.0030 (0.9963–1.0061)	0.9885–1.0330	0.021	Non-normal

Note. ROI-L = lesion ROI; nROI = ROI-L/ROI-P; fROI = ROI-L/ROI-F; mROI = ROI-L/ROI-M; SD = standard deviation; IQR = interquartile range. Continuous variables are reported using both mean ± SD and median (IQR) for descriptive purposes. ROI size is expressed in cm^2. Analyses were performed on 27 lesion-level observations for each ROI size and acquisition phase.

Interpretation. Several absolute and normalized ROI-derived parameters showed non-normal or borderline distributions, particularly in delayed-phase measurements. These findings justify reporting central tendency mainly as median (IQR) and using non-parametric tests in the main comparative analyses.
